# Supplementary material for: Prediction of Adrenocortical Carcinoma Relapse and Prognosis with a Set of Novel Multigene Panels
Source: Cancers (Basel). 2022 Jun 5;14(11):2805. doi: 10.3390/cancers14112805 (PMC9179637; doi:10.3390/cancers14112805)
Supplement: Supplementary file 1 [file cancers-14-02805-s001.zip › Figure S1.pdf]

Supplementary Figure S1

A

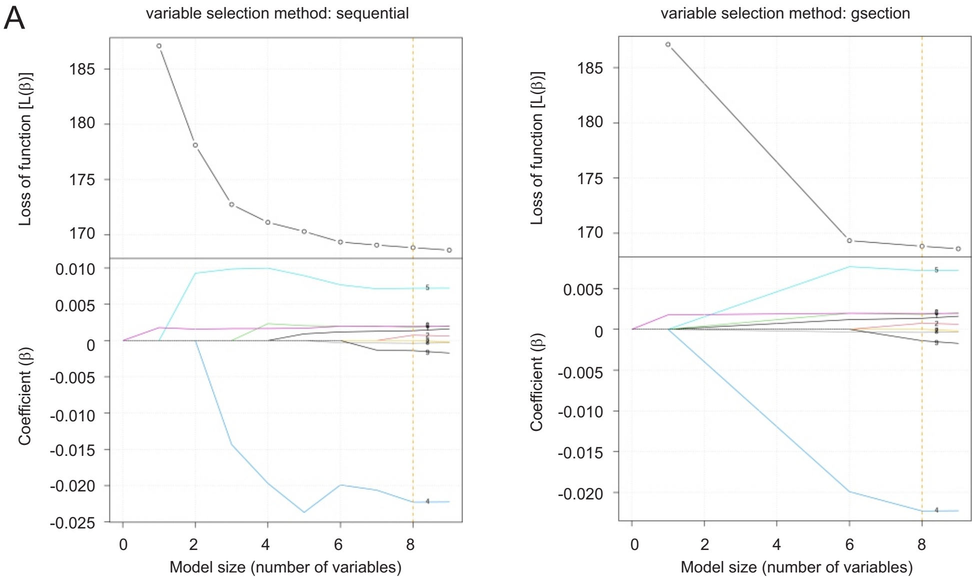

B

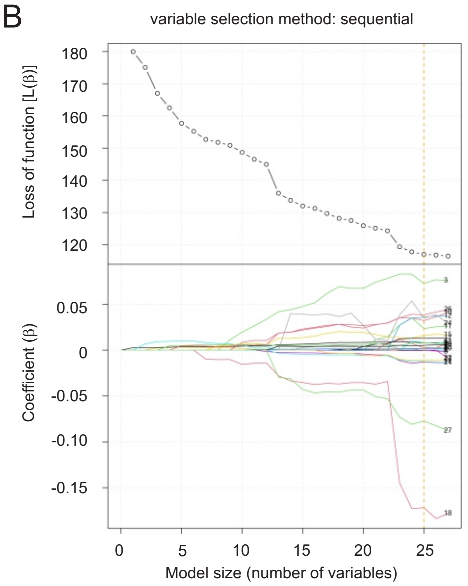

Figure S1: Optimization of ccRCC SigIQGAP1NW and PC Sig27gene multigene panels in predicting ACC fatality. (A) Determination of model (Signature) size of SigIQGAP1NW using Cox regression within the BeSS R package using both sequential (left panel) and golden selection (gselection) (right panel) methods. (B) Optimization of Sig27gene with BeSS using the sequential method.
