# Supplementary material for: Prediction of Adrenocortical Carcinoma Relapse and Prognosis with a Set of Novel Multigene Panels
Source: Cancers (Basel). 2022 Jun 5;14(11):2805. doi: 10.3390/cancers14112805 (PMC9179637; doi:10.3390/cancers14112805)
Supplement: Supplementary file 1 [file cancers-14-02805-s001.zip › Figure S2.pdf]

Supplementary Figure S2

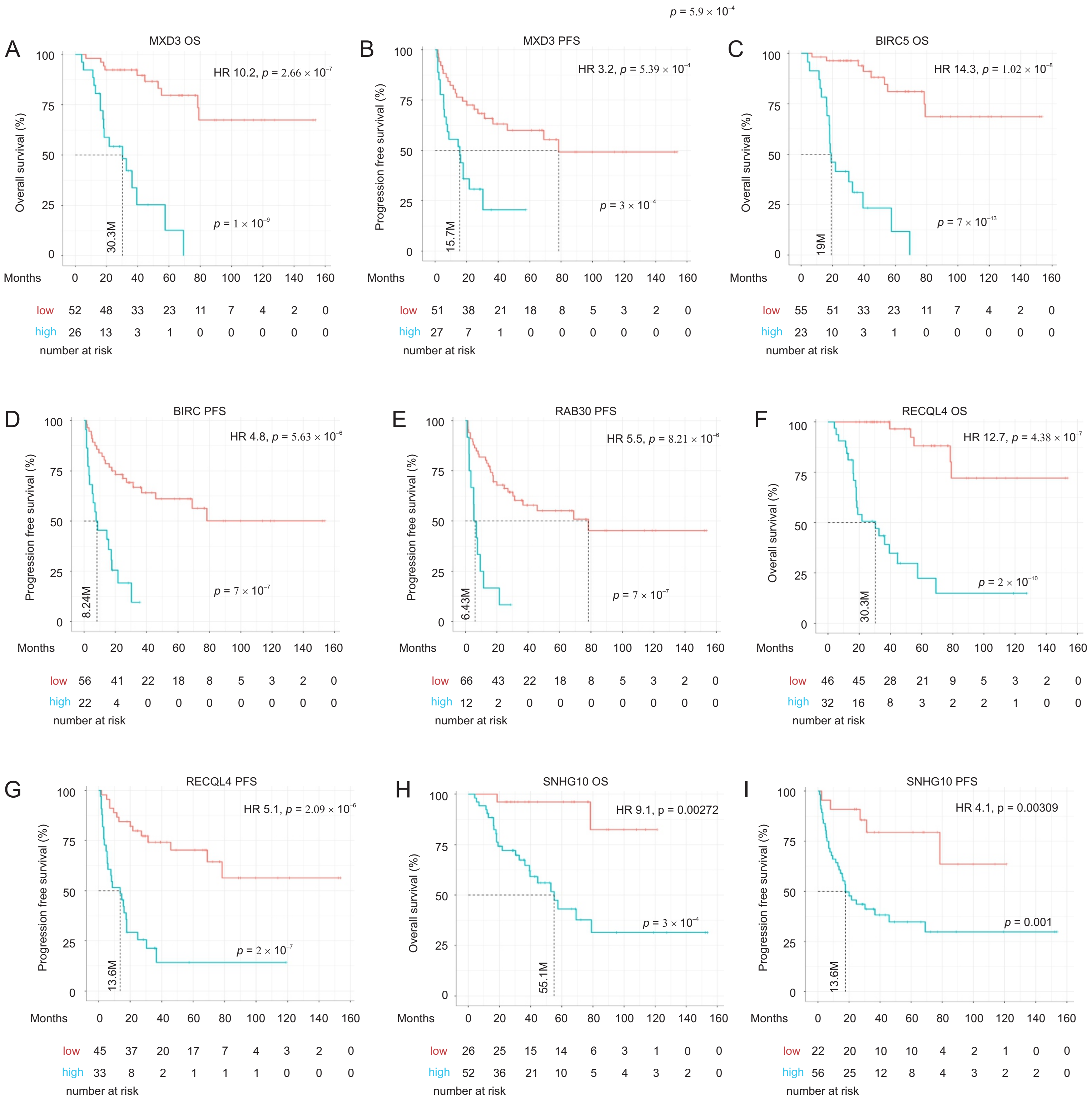

Figure S2: Stratification of ACC progression and fatality risk by MXD3, BIRC5, RAB30, RECQL4, and SNHG10. Cutoff points were estimated on individual gene expression using Maximally Selected Rank Statistics (the Maxstat package) in R. Statistical analyses were performed with logrank test. HRs for the risk of ACC progression and poor prognosis in the stratified groups were derived using univariate Cox analysis with the R survival package.
