# Supplementary material for: Prediction of Adrenocortical Carcinoma Relapse and Prognosis with a Set of Novel Multigene Panels
Source: Cancers (Basel). 2022 Jun 5;14(11):2805. doi: 10.3390/cancers14112805 (PMC9179637; doi:10.3390/cancers14112805)
Supplement: Supplementary file 1 [file cancers-14-02805-s001.zip › Figure S3.pdf]

Supplementary Figure S3

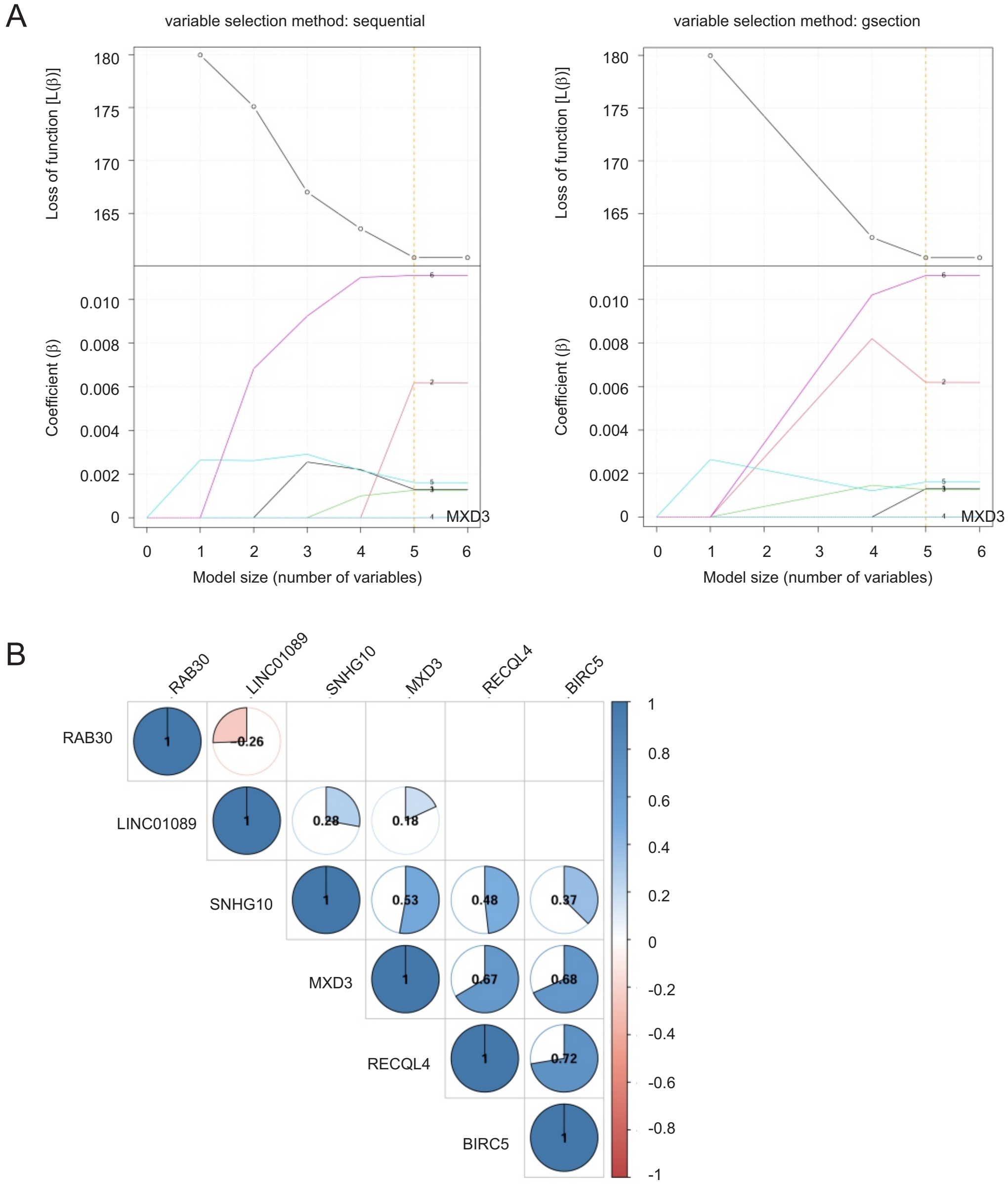

Figure S3: Signature size selection for SigCmbnvar5. (A) The combination of 6 genes from SigIQvar8 and Sig27var25 (LINC01089, SNHG10, RECQL4, MXD3, BIRC5, and RAB30) were selected for the best panel to predict ACC poor prognosis using the Cox model within BeSS. Selections were performed using the sequential (left) and gsection (right) method. (B) Spearman correlation of the indicated genes. Correlations with $p < 0.01$ are included; specific correlation coefficient is also shown.
