# Supplementary material for: Prediction of Adrenocortical Carcinoma Relapse and Prognosis with a Set of Novel Multigene Panels
Source: Cancers (Basel). 2022 Jun 5;14(11):2805. doi: 10.3390/cancers14112805 (PMC9179637; doi:10.3390/cancers14112805)
Supplement: Supplementary file 1 [file cancers-14-02805-s001.zip › Figure S4.pdf]

Supplementary Figure S4

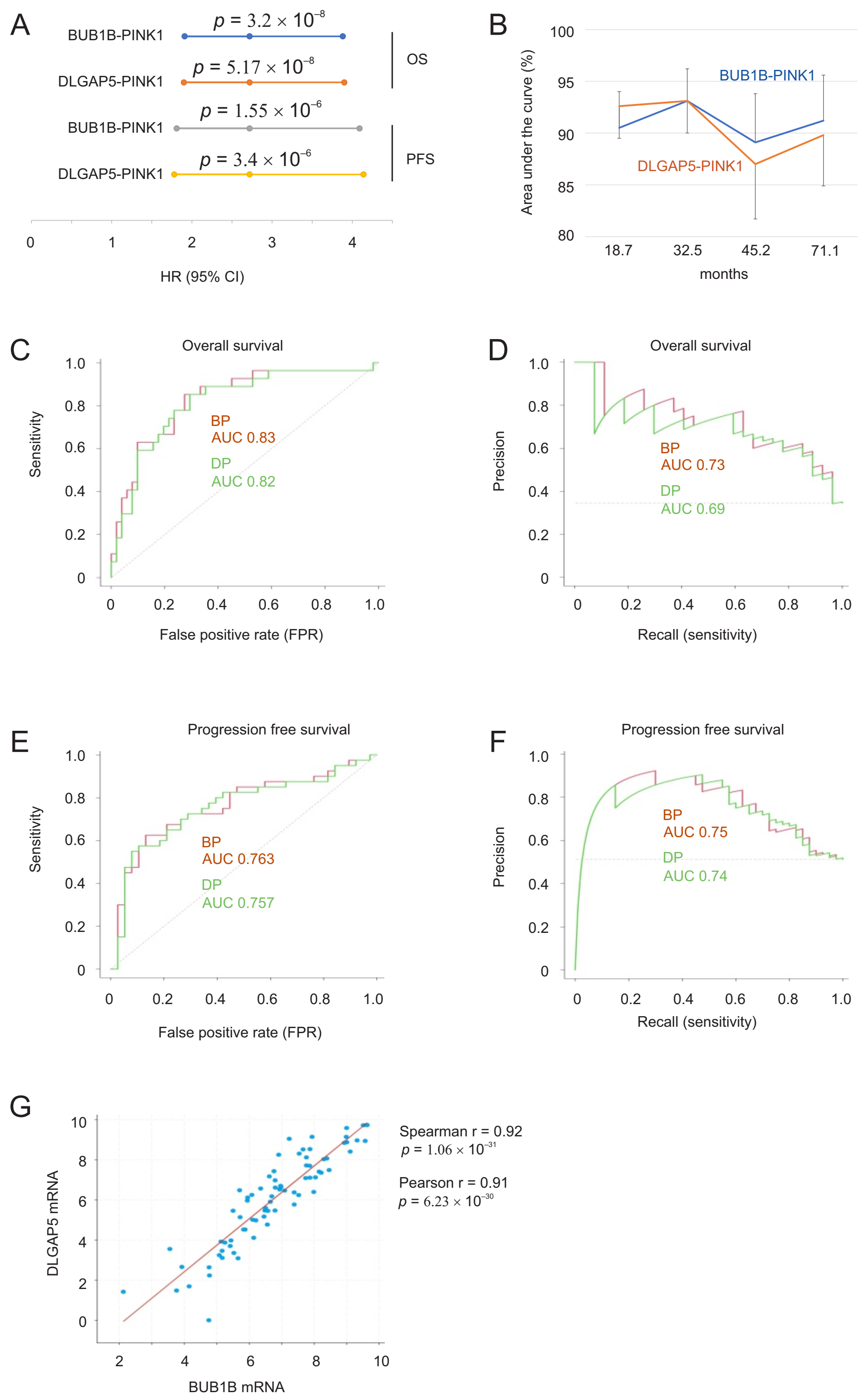

Figure S4: Evaluation of the biomarker potential of two public signatures: BUB1B_PINK1 and DLGAP5_PINK1. (A) Forestplot for HR, 95% CI, and p values for BUB1B_PINK1 and DLGAP5_PINK1 in predicting poor OS and worse PFS. (B) Time-dependent ROC-AUC curves for the indicated panels in stratification of poor OS. (C, D) ROC-AUC and PR-AUC curves for the indicated signatures in discrimination of poor OS. (E, F) ROC-AUC and PR-AUC curves for the indicated signatures in stratification of worse PFS. (G) Correlations between BUB1B and DLGAP5 mRNA expression in the TCGA pan-cancer ACC dataset were produced using tools provided by cBioPortal.
