# Supplementary material for: Prediction of Adrenocortical Carcinoma Relapse and Prognosis with a Set of Novel Multigene Panels
Source: Cancers (Basel). 2022 Jun 5;14(11):2805. doi: 10.3390/cancers14112805 (PMC9179637; doi:10.3390/cancers14112805)
Supplement: Supplementary file 1 [file cancers-14-02805-s001.zip › Figure S5.pdf]

Supplementary Figure S5

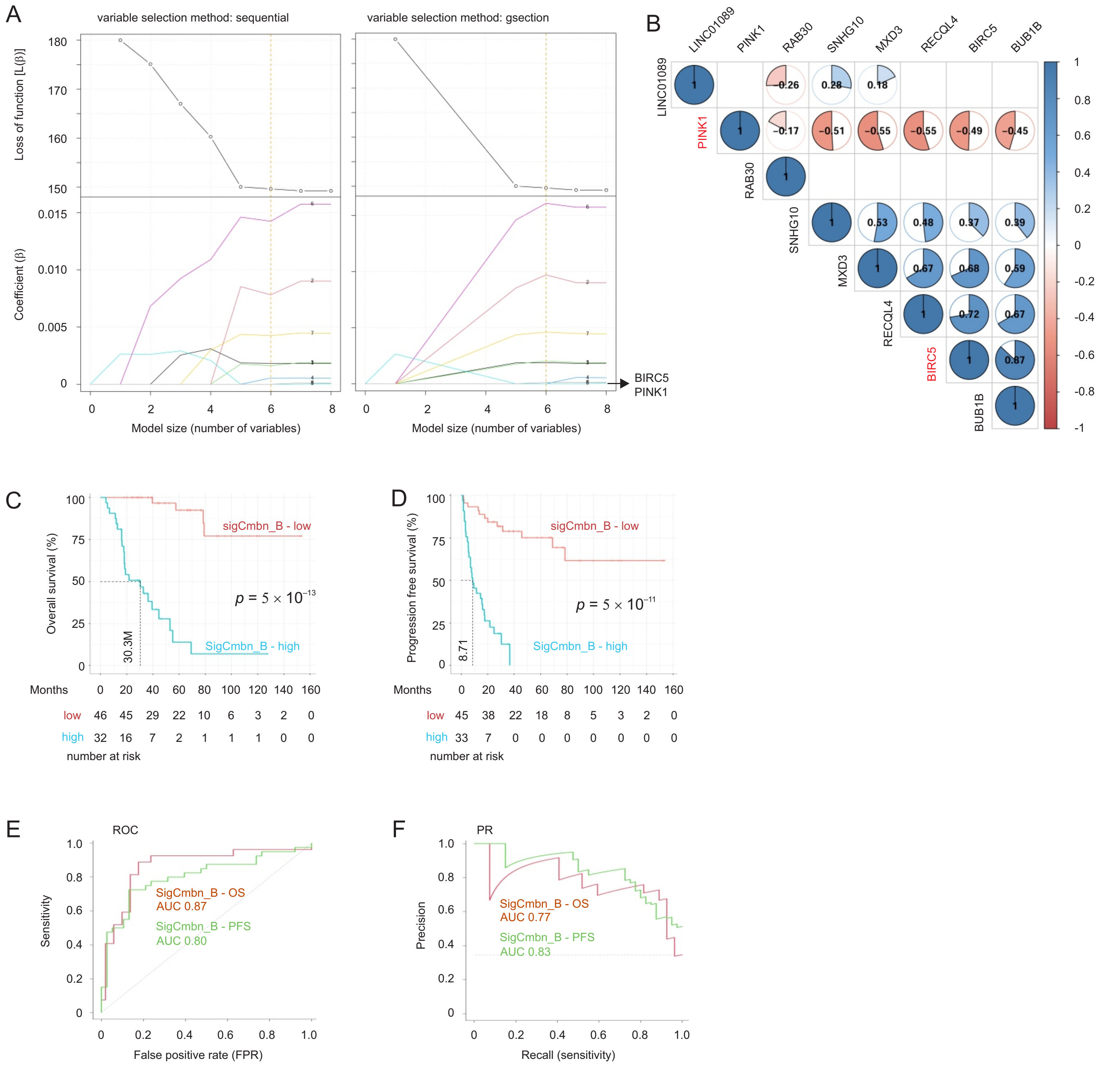

Figure S5: Construction and characterization of SigCmbn_B. (A, B) The combination of 6 genes from SigIQvar8 and Sig27var25 (LINC01089, SNHG10, RECQL4, MXD3, BIRC5, and RAB30) and 2 published genes (BUB1B and PINK1) were selected for the best panel to predict ACC poor prognosis using the Cox model within BeSS. Selections were performed using the sequential (left) and gsection (right) method; both BIRC5 and PINK1 are excluded (A). Spearman correlation of the indicated genes. Correlations with $p < 0.01$ are included; specific correlation coefficient is also shown. Both PINK1 and BIRC5 are eliminated and other 6 genes are in SigCmbn_B panel. (C, D) Stratification of poor OS and worse PFS by SigCmbn_B; cutoff points were estimated by the empirical method. (E, F) ROC and PR curves for SigCmbn_B in discrimination of poor OS and worse PFS.
