# Supplementary material for: Prediction of Adrenocortical Carcinoma Relapse and Prognosis with a Set of Novel Multigene Panels
Source: Cancers (Basel). 2022 Jun 5;14(11):2805. doi: 10.3390/cancers14112805 (PMC9179637; doi:10.3390/cancers14112805)
Supplement: Supplementary file 1 [file cancers-14-02805-s001.zip › Figure S6.pdf]

Supplementary Figure S6

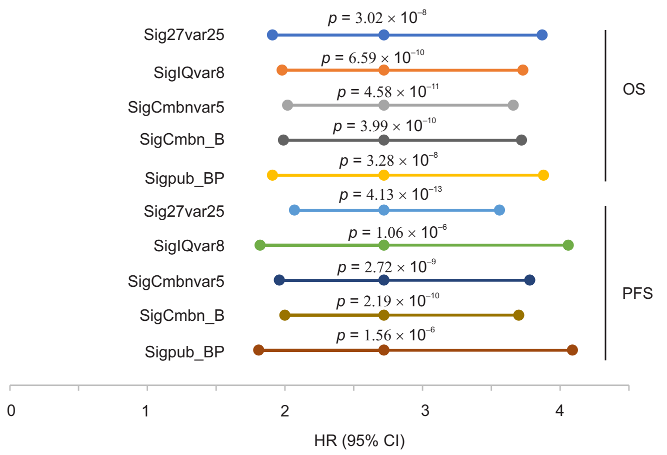

Figure S6: HR (hazard ratio) for Sig27var25, SigIQvar8, SigCmbnvar5, SigCmbn_B, and Sigpub_PB in predicting poor OS and worse PFS.
