# Supplementary material for: Prediction of Adrenocortical Carcinoma Relapse and Prognosis with a Set of Novel Multigene Panels
Source: Cancers (Basel). 2022 Jun 5;14(11):2805. doi: 10.3390/cancers14112805 (PMC9179637; doi:10.3390/cancers14112805)
Supplement: Supplementary file 1 [file cancers-14-02805-s001.zip › Figure S7.pdf]

Supplementary Figure S7

A

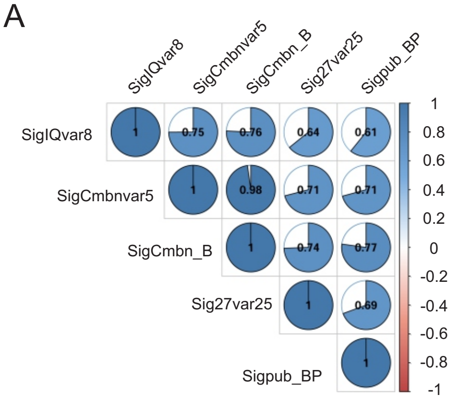

B

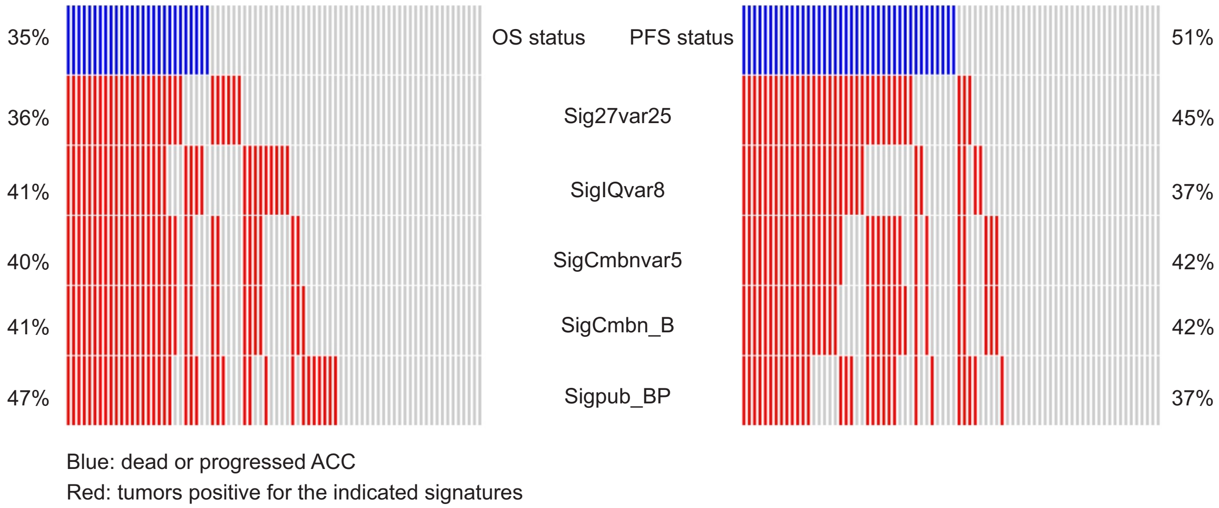

Figure S7: Correlations of Sigpub_BP with our gene panels (Sig27var25, SigIQvar8, SigCmbnvar5, SibCmbn_B). (A) The indicated correlations were determined using Spearman correlation. (B) Heatmap was constructed using the “oncoprint” function in the ComplexHeatmap R package [30]. Dead and progressed tumors are marked with blue. ACC positive for the indicated signatures are colored red. Positive and negative tumors were defined according to the respective cutoff points estimated by empirical method. Percentages for the positive events are indicated.
