# Supplementary material for: Prediction of Adrenocortical Carcinoma Relapse and Prognosis with a Set of Novel Multigene Panels
Source: Cancers (Basel). 2022 Jun 5;14(11):2805. doi: 10.3390/cancers14112805 (PMC9179637; doi:10.3390/cancers14112805)
Supplement: Supplementary file 1 [file cancers-14-02805-s001.zip › Figure S8.pdf]

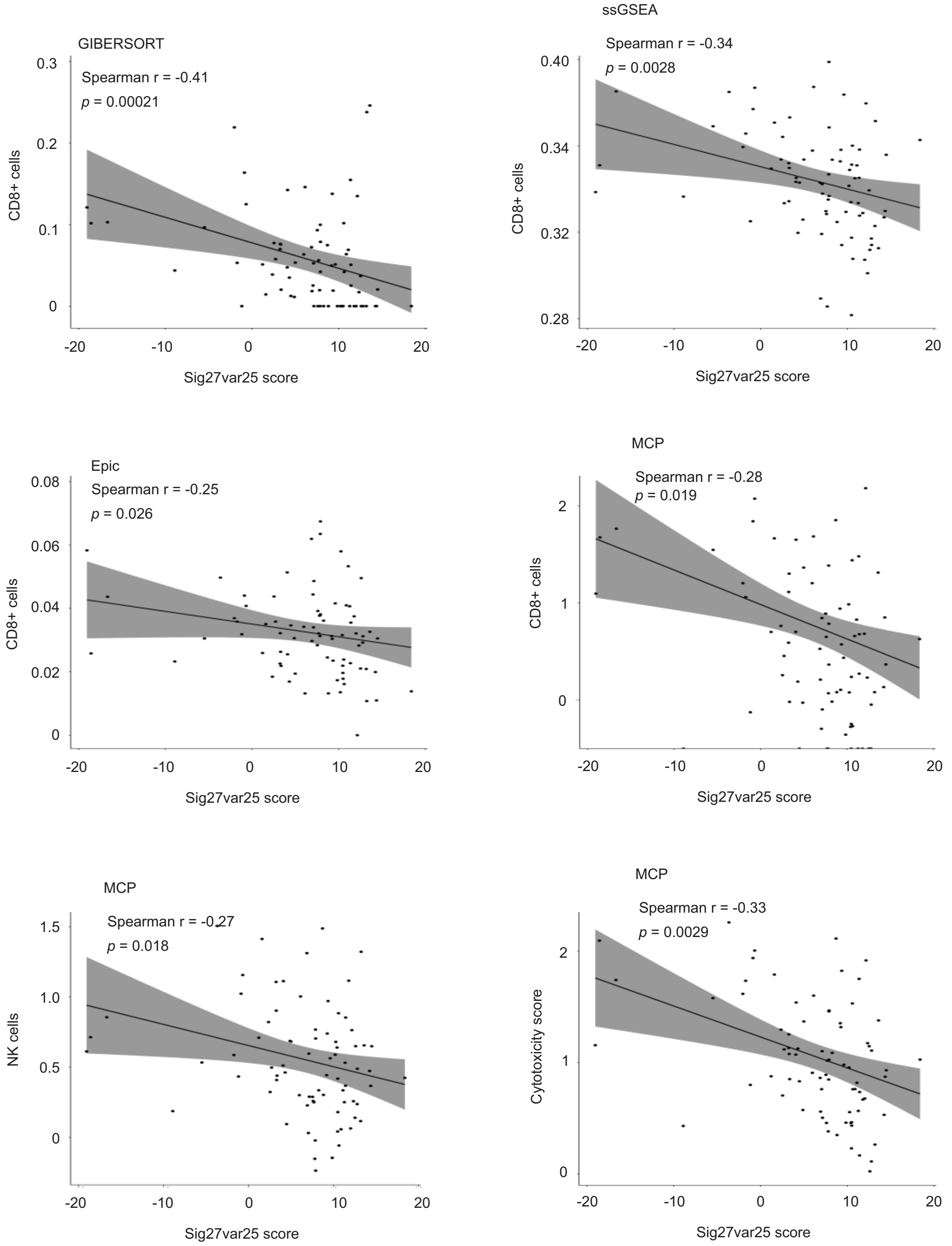

Figure S8: Correlations of Sig27var25 scores with CD8+, NK, and cytotoxic scores. The programs used to produce these correlations are indicated, i.e. GIBERSORT, ssGSEA, Epic, and MCP.
